# Analysis of the Global Warming Potential of Biogenic CO_2_ Emission in Life Cycle Assessments

**DOI:** 10.1038/srep39857

**Published:** 2017-01-03

**Authors:** Weiguo Liu, Zhonghui Zhang, Xinfeng Xie, Zhen Yu, Klaus von Gadow, Junming Xu, Shanshan Zhao, Yuchun Yang

**Affiliations:** 1 School of Natural Resources, West Virginia University, Morgantown, WV 26506, United States; 2 Jilin Province Academy of Forestry Research, Changchun, 130033, China; 3 School of Forest Resources and Environmental Science, Michigan Technological University, Houghton, MI 49931, United States; 4 Department of Ecology, Evolution, and Organismal Biology (EEOB), Iowa State University, Ames, IA 50011, United States; 5 Burckhardt Institute, Georg-August University Göttingen, Göttingen, Germany; 6 Institute of Chemical Industry of Forest Products CAF, Nanjing, Jiangsu, China

## Abstract

Biomass is generally believed to be carbon neutral. However, recent studies have challenged the carbon neutrality hypothesis by introducing metric indicators to assess the global warming potential of biogenic CO_2_ (GWP_bio_). In this study we calculated the GWP_bio_ factors using a forest growth model and radiative forcing effects with a time horizon of 100 years and applied the factors to five life cycle assessment (LCA) case studies of bioproducts. The forest carbon change was also accounted for in the LCA studies. GWP_bio_ factors ranged from 0.13–0.32, indicating that biomass could be an attractive energy resource when compared with fossil fuels. As expected, short rotation and fast-growing biomass plantations produced low GWP_bio_. Long-lived wood products also allowed more regrowth of biomass to be accounted as absorption of the CO_2_ emission from biomass combustion. The LCA case studies showed that the total life cycle GHG emissions were closely related to GWP_bio_ and energy conversion efficiency. By considering the GWP_bio_ factors and the forest carbon change, the production of ethanol and bio-power appeared to have higher GHG emissions than petroleum-derived diesel at the highest GWP_bio_.

Biomass is generally considered as a carbon neutral energy resource because the emissions from biomass combustion will be absorbed by plants through photosynthesis^1^,^2^. The first comprehensive guideline for estimating greenhouse gas (GHG) emissions and sinks alleged that “CO_2_ emission resulting from bioenergy consumption should not be included in a country’s official emission inventory”^3^. According to the guidelines compiled by the Intergovernmental Panel on Climate Change (IPCC), CO_2_ emission from bioenergy sources should not be counted in national greenhouse gas inventories because the emission from bioenergy sources is already fully included in the Agriculture, Forestry and Other Land-Use (AFOLU) sector^4^. Therefore, bioenergy is always referred as a carbon neutral source of energy and promoted by government policies as a substitute for fossil fuels. For the same reason, no carbon tax is applied for burning biomass in any country around the world.

Due to the assumption of zero climate change potential of biomass combustion, the methods for assessing its global warming impact in environmental analysis tools (e.g. SimaPro, GaBi) usually do not include the biogenic CO_2_ emission and even treat biogenic CO_2_ emission as a negative impact^5^,^6^,^7^. In addition, most of the life cycle assessment (LCA) studies conducted on bioenergy systems also claim that CO_2_ emission from biomass has no global warming potential (GWP). Cherubini and Strømman reviewed 94 LCA studies of bioenergy systems and found that only one single case study included an account of the climate change impact of biogenic CO_2_ emission^8^. Shonnard *et al*. reviewed 74 LCA case studies in the Pan American region and found that most of the articles assumed carbon neutrality of biomass combustion^9^. This carbon neutrality assumption, which could reduce the GWP of a bioenergy system, might be suitable if the rotation length of biomass were as short as in the case of perennial grass. However, the assumption may not hold when the rotation lengths are long, especially such as those of boreal forests which can be 100 years^10^. The exclusion of biogenic CO_2_ emission in LCA also brings an unfair comparison of the GHG emissions from bioenergy and fossil fuel systems, although biogenic CO_2_ emission is fully accounted for in the AFOLU sector.

More recently, researchers have become aware that CO_2_ emission from biomass combustion may have, to some extent, a climate change impact because the CO_2_ emitted by biomass combustion is a one-time pulse and remains in the atmosphere for several years. Johnson strongly asserted that biomass fuel might not always be carbon neutral and could in fact have a far greater impact than fossil fuels^11^. Searchinger *et al*. also questioned the carbon neutrality assumption and suggested that shorter rotation lengths would be able to lower the GWP by absorbing the emissions after biomass combustion^12^. Several recent studies have proposed a method to calculate the GWP of CO_2_ emission derived from biomass, providing estimates of GWP_bio_ factors for different scenarios^8^,^10^,^13^,^14^,^15^. The GWP_bio_ was calculated by the relative radiative forcing effect during its stay in the atmosphere. With a 100-year time horizon, their estimates of GWP_bio_ fell in the range of 0.34–0.62 when the rotation length was 100 years. Cherubini *et al*. estimated GWP_bio_ for rotation lengths from 1to 100 years and revealed a negligible GWP_bio_ for short rotation lengths^10^. If the forest stands were harvested for long-lived wood products, the GWP_bio_ could be as low as -1if the wood products were collected for bioenergy after decommissioning^13^.

The application of GWP_bio_ factors is straight forward. These factors can be considerably lower than 1, implying an advantage of bioenergy compared to fossil fuels in a climate change perspective. However, Holtsmark articulated the weakness of the previous methods which neglected the effect of harvesting on the dynamics of the major carbon pools in forest stands, such as logging residues and soil carbon^16^. He defined a no residue harvest baseline scenario and determined the GWP_bio_ factors of other scenarios by comparing them to this baseline. The derived GWP_bio_ in a 100-year time horizon was 1.54 for no residue harvested and 1.25 for 25% of residues harvested. In another study, Holtsmark included the albedo effect by collecting residues, which slightly reduced GWP_bio_^17^.

These earlier studies provided fundamental results to bring a fair comparison of CO_2_ emissions from bioenergy and fossil fuel. However, two considerations need to be clarified for a more accurate accounting: 1) the residue, if not collected, will decompose slowly and emit CO_2_ into the atmosphere over the following years, and 2) the harvest of biomass or timber will result in further emissions from dead organic matter. Thus, a method to estimate GWP_bio_ accurately should address these considerations. In our study, we analyzed the GWP_bio_ by applying an accounting model considering long-lived wood products and the decomposition of wood residues. The GWP_bio_ factor also applied to five LCA studies of bioproducts to estimate the potential of biomass utilization in reducing carbon emissions.

## Materials and Methods

### Forest Stand Modeling

A forest stand storing 80 tC/ha live biomass was harvested at the start (t = 0). The biomass accumulation after harvesting was estimated by a Chapman-Richards function: 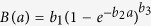, where *B(a)* is the mass of biomass measured in tC/ha (tC: metric ton carbon equivalent) and *a* the stand age; the *b*_*i*_ are empirical parameters assumed to be known. The harvest activity resets all the living biomass to zero. This function provides a reasonable growth of the stand as a function of its age. Three sets of parameter configurations were used to allow the stand to grow back to its previous carbon level after harvesting in 30, 50 and 100 years (Table 1).

The average merchantable timber is assumed to be a proportion of live biomass. We are using the proportion *θ* = 0.48 proposed by Løken *et al*.^19^. The merchantable timber is thus calculated as: *T(a*) = *θB(a*), where *T(a)* is the merchantable timber and *B(a)* is live biomass. The unit of *T(a)* and *B(a)* is metric ton in carbon equivalent (tC). In this study, we assumed a proportion *ω* = 0.25 of residues is collected for bioenergy. The biomass not harvested is considered to be dead organic matter (DOM). The decomposition process is simulated by Yasso07 with average amounts of compartments in the residue (See Table S1 in the supporting information). Yasso07 is a widely-used model for simulation of biomass decomposition^20^. The decomposition rate is shown in Fig. S1.

### Biogenic CO_2_ in the Atmosphere

Once fossil fuel derived CO_2_ is emitted to the atmosphere, the decay rate is not following a simple trajectory. The fraction of the initial pulse of CO_2_ at time *t* is labeled as *y(t)* and calculated as follows:


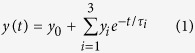


where *y*_*i*_ and *τ*_*i*_ are estimated parameters. This model is based on the Bern2.5CC carbon cycle model using a CO_2_ concentration of 378 ppm in the atmosphere^21^. The parameters are average values of a set of climate models and set as *y*_0_ = 0.217, *y*_1_ = 0.224, *y*_2_ = 0.282, *y*_3_ = 0.276, *τ*_1_ = 394.4, *τ*_2_ = 36.54, *τ*_3_ = 4.304^21^. Equation (1) takes into account the CO_2_ uptake from the oceans and the terrestrial biosphere. Because *y*_0_ > 0, there is always a portion of CO_2_ remaining in the atmosphere at any time.

When residue is collected for bioenergy, the initial pulse of CO_2_ is *E(ω*) = *ω(B(a*_*h*_*) − T(a*_*h*_)), where *a*_*h*_ is the stand age at harvest. After harvesting, stand age *a* equals *t*. Let *E*_*h*_*(t)* as CO_2_ remains in the atmosphere after the pulse of *E(ω)* at time *t*, and *E*_*d*_*(t)* as CO_2_ emission from decomposition at time *t* if the residue is not collected. Hence, we have the following equations:


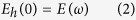



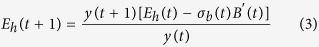



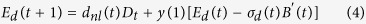


where *σ*_*b*_(*t*) and *σ*_*d*_(*t*) are mass allocation coefficients for the residue if the residue is used for bioenergy or is not removed respectively. *B*′(*t*) is the increment of live biomass after harvesting. Because the stand is harvested for timber and these timber products usually do not emit CO_2_ to the atmosphere immediately, we consider two scenarios. In the first case, the timber products have a pulse of CO_2_ right after harvesting (*η*_*l*_ = 0%); in the other case the timber products do not emit any CO_2_ before the end of the rotation (*η*_*l*_ = 100%). *η*_*l*_ is percentage of long-lived woody products in the total merchantable timber. The detailed calculation of the mass allocation coefficients are presented in the supporting information. *E*_*h*_(*t*) − *σ*_*b*_(*t)B*′(*t*) and *E*_*d*_(*t*) − *σ*_*d*_(*t)B*′(*t*) will be set to zero once these value are lower than zero because CO_2_ absorbed by biomass regrowth can fully compensate for the CO_2_ remaining in the atmosphere. If the residues are not collected, they decompose and emit CO_2_ into the atmosphere. Thus, the emission from decomposition is subtracted and the CO_2_remaining in the atmosphere at time *t* is as follows:


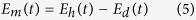


### Global Warming Potential

The Global Warming Potential is used in this study as a standard estimation to compare the climate change impact of GHG emissions. The GWP is introduced as a relative measure by comparing the amount of heat that a greenhouse gas traps in the atmosphere to the amount of heat trapped by a similar mass of CO_2_. The GWP relies heavily on radiative forcing where the radiative forcing of CO_2_ (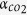) is 1.37 × 10^−5^ W m^−2^ ppb^−1^ by Myhre *et al*.^22^. The absolute global warming potential (AGWP) of a CO_2_ pulse of *E(ω*) is calculated as follows:


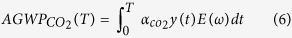


where *T* is the time horizon for integration in the GWP. The IPCC introduced three time horizons for this purpose, i.e., 20, 100 and 500 years. In this study, we considered *T* = 100 as the time horizon which is generally used to estimate the overall GHG emissions in most LCA studies.

Similarly, the AGWP_bio_ is calculated based on the remaining fraction of the initial pulse of CO_2_ by subtracting the decomposition of DOM. The AGWP_bio_ is:


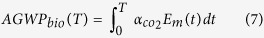


and the GWP_bio_ is then defined as:


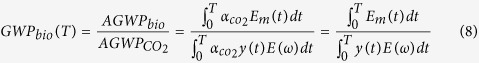


### LCA Case Studies

In order to analyze the effect of this adjusted GWP_bio_ factor, five biomass to bioproducts pathways were tested: biomass to ethanol (BTE), biomass to liquid fuel via fast pyrolysis (BLFP), coal and biomass to liquids (CBTL), biomass to bio-power and biomass to pellet fuel. The cradle-to-grave assessments included residue collection, transportation, storage, preprocessing, bioproduct conversion, distribution, final usage and waste disposal. These studies focused on GHG emissions derived from fossil fuels and biomass combustion. The functional unit (FU) of the system was 1,000 MJ of energy equivalent bioproduct produced. The GHG emissions of petroleum-derived diesel with 98.8 kg CO_2_ eq/FU were used as a baseline to determine the advantage of biomass on climate change impact^23^. The impact of GHGs was calculated using 100-year global warming potentials^19^. All the GHG emissions were calculated in CO_2_ equivalent (kgCO_2_ eq). The system boundaries are defined in the Supplemental Information and the detailed life cycle inventory data are also available in Supplemental Information^24^. Traditional LCA quantifies the emissions related to the production of bioproduct and the harvest of biomass. Several researchers suggest that emissions related to land use change and forest carbon change should be included^25^,^26^. Although the forest stand in this study is allowed to regrow and the land use does not change, forest carbon change induced by biomass collection was accounted for in the LCA studies. See supporting information for details regarding the method of calculating forest carbon change.

## Results

### GWP_bio_ factors

**Figure 1:**
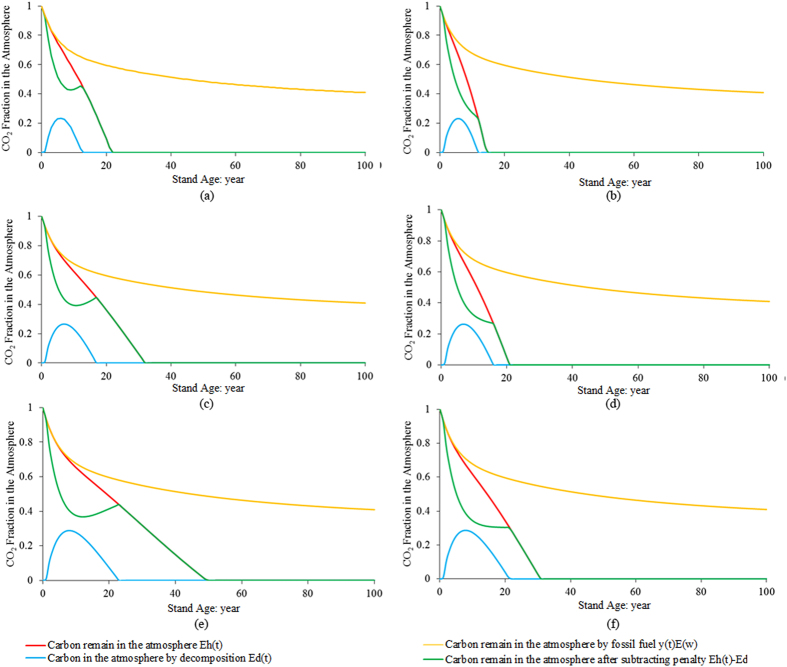
Carbon decay patterns for different scenarios. (**a**) Rotation length 30-year, *η*_*l*_ = 0%; (**b**) Rotation length 30-year, *η*_*l*_ = 100%; (**c**) Rotation length 50-year, *η*_*l*_ = 0%; (**d**) Rotation length 50-year, *η*_*l*_ = 100%; (**e**) Rotation length 100-year, *η*_*l*_ = 0%; (**f**) Rotation length 30-year, *η*_*l*_ = 100%.

As explained in the methods section, we examined two scenarios: 1) *η*_*l*_ = 0% where the timber products emit CO_2_ at the same time as biomass combustion, and 2) *η*_*l*_ = 1000% indicated all timber products do not emit CO_2_ before the end of the rotation. Compared to fossil fuel derived CO_2_ which decays with the interaction of ocean-atmosphere systems, these scenarios allow an increasing decay rate of biogenic CO_2_ in the atmosphere if the harvested stand is re-established and allowed to regrow (Fig. 1).

In Fig. 1, *E*_*h*_*(t), E*_*d*_*(t)* and *y(t)E(w)* are the amount of CO_2_remaining in the atmosphere from biomass combustion, decomposition and fossil fuel, respectively. The curve representing *E*_*h*_*(t)-E*_*d*_*(t)* is not a decay curve but the curve that we used to calculate GWP_bio_. Higher values of σ (representing a longer lifespan of timber products) allow higher decay rate of biogenic CO_2_ (Fig. 1b,d,f). Higher biomass growth rate could also effectively increase the decay rate of biomass derived CO_2_ (Fig. 1). It took 21, 32 and 49 years, assuming *η*_*l*_ = 0%, to eliminate all the biomass derived CO_2_ when the rotation length was 30, 50 and 100 years. If *η*_*l*_ = 100%, the corresponding periods were 14, 20 and 30 years.

The GWP_bio_ factors in this study were significantly lower than 1 and related to the rotation length and the mass allocation coefficient (Table 2). All our assumptions led to lower GWP_bio_ factors when compared to earlier studies, especially when wood products have a long lifespan (Table 2). Sensitivity analyses were conducted on rotation length and η_1_. The GWP_bio_ varied between 0.13 and 0.32 as the rotation length changed from 30 years to 100 years (Fig. S3). The GWP_bio_ was linearly related to η_1_ as it changed from 0% to 100%.

The model allowed the modification that the CO_2_ absorbed in previous years was excluded from the atmosphere over the following years. The model also subtracted the emission from the decomposition of residues if they were not harvested. Decomposition was considered to be slow but could have a positive global warming impact in a young stand where the biomass accumulation is slow (Table S3). These modifications allowed a logical explanation of the benefits from regrowth of a forest stand.

### GHG emissions of Case Studies

**Figure 2:**
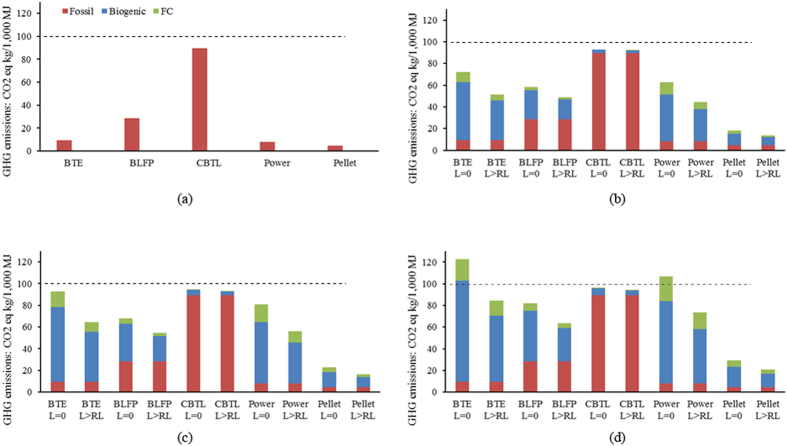
GHG emissions of biomass to bioproduct pathways under different scenarios. (**a**) no biogenic CO_2_ emission; (**b**) RL = 30; (**c**) RL = 50 and (d) RL = 100. Note: Fossil – GHG emissions from fossil fuel; Biogenic – accountable biogenic GHG emissions from biomass; FC – accountable forest carbon change; RL – Rotation length.

Without considering biogenic CO_2_ emission, all five bioproducts had lower GHG emissions in comparison with petroleum-derived diesel (Fig. 2a). The production of pellet fuel had the lowest GHG emissions, at only 4.8% of petroleum-derived diesel. The production of ethanol and bio-power also produced low GHG emissions. Liquid fuels produced by fast pyrolysis had higher GHG emissions compared to the production of ethanol, bio-power and pellet fuel. The highest emissions were estimated from CBTL, amounting to 90% of the emissions from petroleum-derived diesel.

When considering the biogenic CO_2_ emission in LCA, we found that most of the biogenic CO_2_ emission was produced in “conversion” and “final usage” (Table 3). All the energy usage in “feedstock collection”, “transportation” and bioproduct “distribution” was assumed to be provided from fossil energy. In the production of bio-power in particular, 99% of the total biogenic CO_2_ was accounted for by emission in the “conversion” process. In the production of bio-power, all biomass was combusted at the facility site and no further emissions were assumed in the end usage.

The GHG emissions from ethanol and bio-power were very low compared to other bioproducts if the GWP_bio_ factor was zero, but both appeared to have high GHG emissions when the rotation length was 100 years and *η*_*l*_ = 0%. When forest carbon change was accounted for in the LCA study, both had higher GHG emissions than petroleum-derived diesel. The GHG emissions of CBTL did not exceed those of petroleum-derived diesel in all cases due to the low percentage of biomass required to produce liquid fuels.

Generally, shorter rotation lengths and higher mass allocations benefit the environment more in terms of GHG emissions (Fig. 2b,c,d). To produce the same bioproduct, the GHG emissions were the lowest when the rotation length was 30 years. It was 1.4–41.4% and 2.5–39.2% less than the GHG emissions when the rotation length was 50 years and 100 years, respectively. To produce the same bioproduct, the GHG emissions when *η*_*l*_ = 0% were 1–25% higher than the GHG emissions in the *η*_*l*_ = 100% scenario in all cases.

## Discussion

### GWP_bio_ factors

Our model assumed that the primary objective of harvesting is for timber products which usually have a longer lifespan than biomass. It was also assumed that the dynamics of soil carbon would not be affected by a small percentage (≤25%) of residue removal. The portion of biomass regrowth accounted for CO_2_ absorption depended on the percentage of CO_2_ remaining in the atmosphere from biomass combustion. We examined two mass allocation scenarios. If the wood products emitted CO_2_ before the end of the rotation, the mass allocation coefficient would fall between these two scenarios. A long lifespan of timber products allows a high mass allocation coefficient, and short rotations imply rapid regrowth of a forest stand. Thus, the decay rate of biogenic CO_2_ increases with decreasing rotations and increasing lifespan of timber products. The forest carbon change due to harvesting was attributed to timber and biomass collection and allocated based on their mass.

The GWP_bio_ factors in all scenarios are significantly lower than 1. This indicates the advantage of biomass when compared to fossil fuels. An even lower GWP_bio_ factor would be possible if growth would be more vigorous at a younger age of the forest stand. The GWP_bio_ factors correlates with rotation length, but this relationship should not be over exploited. It merely implies that higher growth rates may accelerate the decay of CO_2_ in the atmosphere. In reality, the rotation length of a forest stand is relatively long. For fast growing tree species such as eucalypts, the rotation length is at least 15–18 years^27^,^28^. The pulse of CO_2_ from biomass combustion can be neglected in the case of perennial grasses which rotation length is 1 or 2 years^10^,^29^.

The high GWP_bio_ factor in Holtsmark’s results could be reasonable if, as assumed in his study, all the trunks and harvested residues were used for biomass combustion. This suggests that all the changes of major carbon pool in the stand should be accounted for by biomass combustion. Nonetheless, in most cases stands are harvested for timber. In Cherubini *et al*.’s study, the forest model was modified to allow more vigorous growth in younger stands leading to slightly lower GWP_bio_ factors^10^,^17^. Recent estimates of GWP_bio_ factors may be too optimistic because no negative effects on the growth of biomass were included, like natural disturbances and climate change. Natural disturbance, like wild fire, drought, insect and pathogen outbreaks, will reduce biomass growth and even induce more emission to the atmosphere^30^. These negative effects will increase the possibility of high GWP_bio_ factors, but relevant scenarios may be more realistic for research and policy decisions. The impact of climate change on the growth of biomass is complex. Both negative effects^31^,^32^ and positive effects^33^ were found in earlier investigations. Moreover, harvest level may also be increased for biomass because of high energy demands in the future, which in turn will lead to a higher GWP_bio_ factor.

The GWP_bio_ factors were not calculated for longer time horizon (>100 years), but previous studies have shown that these values may be even lower^10^,^16^. Thus, biomass will be a good substitute of fossil fuels in the long run^10^,^17^,^23^. Growth of biomass can even bring negative GWP if longer time horizons are considered, because biomass will continue to grow and absorb CO_2_. But in this study, the CO_2_ remaining in the atmosphere was set to zero once the increment of biomass growth fully compensates the CO_2_ emission. Part of the reason for that strategy is the assumption that the forest stand absorbs CO_2_ if it is not harvested, which implies that the extra absorption of CO_2_ is not due to residue collection. Additionally, the extra absorption of CO_2_ is external to the studied system (LCA boundary). Considering these CO_2_ reductions may involve some kind of double accounting.

### GHG emissions of Case Studies

Without considering biogenic CO_2_ emission, the life cycle GHG emissions of the five cases closely resembled previous studies^34^,^35^,^36^,^37^,^38^,^39^. The production of pellet fuel had the lowest GHG emissions because of the high energy conversion efficiency and low energy consumption. The reason that the production of ethanol and bio-power had low GHG emissions was the low energy demand in the production of ethanol and the self-sufficiency of power plants on energy. Liquid fuels produced by fast pyrolysis required high electricity input, which led to higher GHG emissions compared to the production of ethanol, bio-power and pellet fuel. CBTL emitted the highest GHG because the large proportion of GHG emissions from the combustion of coal could not be ignored even in conventional LCA studies.

Although the CO_2_ emission from burning biomass is compensated by the growth of biomass, the CO_2_ emission will remain in the atmosphere for a certain time. Thus, it is necessary to consider the GWP of biogenic CO_2_ when conducting LCA. Otherwise, the total GHG emissions will be systematically underestimated. By calculating biogenic CO_2_ in the LCA studies, we found CBTL had a small amount of biogenic CO_2_ emission compared to the other four bioproducts. This is because a small portion of biomass (8%) was needed to produce 1,000 MJ of energy equivalent liquid fuels. Given that biomass was the only feedstock for the other four bioproducts, their biogenic CO_2_ emission were closely related to their energy conversion efficiencies which were 25.6% for BTE^35^, 73.6% for BLFP^36^ and 24% for bio-power^40^. Usually no waste was assumed in the production of wood pellets^41^. By increasing the energy conversion efficiency, biogenic CO_2_ emission could be effectively reduced.

When the biogenic CO_2_ emission was multiplied by a nonzero GWP_bio_ factor, higher GHG emissions were expected in the LCA case studies. This effect was more pronounced when the biogenic CO_2_ emission was high in the production of a bioproduct, such as ethanol and bio-power. The consideration of forest carbon change added more emissions to the total GHG emissions^25^. When a cradle to grave LCA was modeled, the LCI should quantify all the emissions associated with the activities and their effects to produce the product. By adding biogenic CO_2_ emission and forest carbon change, an unbiased comparison could be made between biofuel and fossil fuel, although a significant increase of GHG emissions from biofuel should be expected. However, in this study, the forest carbon change was analyzed at forest stand scale because the timber harvest and residue collection are all occurred in stand scale. In addition, the focus on stand scale fits the objective of this study as well. Here, the study is an attributional LCA model which will not consider effects from outside of the system boundary. Once the study is expanded to landscape level, the forest carbon change could be different.

### Policy Implications

Renewable fuel standard (RSF2) indicates that forest residue makes up less than 1% of the total feedstock under current scenario^42^. Most of the biofuels are produced from corn, soybean, agricultural residue, and grasses. These feedstocks have short rotation period and their GWP of biogenic CO_2_ can be ignored^10^. However, as the renewable fuel volume increases year by year, more forest residue need to be collected for biofuels. To be qualified as renewable energy for cellulosic fuels, the life cycle GHG emissions should be 60% less than the life cycle GHG emissions of the 2005 baseline average gasoline or diesel fuel (92 kg/1,000 MJ)^42^. Thus, the direct influence of GWP_bio_ is that some cellulosic fuel or biopower turn to be non-renewable energy, such as BTE, BLFP and biopower. The other influence is that more short rotation biomass will be used in the future scenarios. Therefore, the GWP_bio_ provides a solid scientific reference to policy makers when making decisions for the utilization of forest residues. However, it is not a simple work to decide the best pathway for forest residue and bioproducts. Many factor should be considered, such as residue availability, cost, market demand, convenience for distribution and usage, GHG emissions and existing facilities. If only GHG emissions are emphasized, pellet fuel is the best choice because it has low emissions in all scenarios.

## Additional Information

**How to cite this article**: Liu, W. *et al*. Analysis of the Global Warming Potential of Biogenic CO_2_ Emission in Life Cycle Assessments. *Sci. Rep.* **7**, 39857; doi: 10.1038/srep39857 (2017).

**Publisher's note:** Springer Nature remains neutral with regard to jurisdictional claims in published maps and institutional affiliations.

## Supplementary Material

Supplementary Information

Supplemental Information

## Figures and Tables

**Table 1:** Parameter settings for the Chapman-Richards function.

Set #	Forest Type	Rotation	*b*_1_	*b*_2_	*b*_3_	Source
1	Tropical rain forest	30 years	428.01	0.0253	2.64	Holtsmark^16^
2	Temperate deciduous forest	50 years	198.6	0.0253	2.64	Htsmark^16^
3	Boreal forest	100 years	103.067	0.0245	2.6925	Asante *et al*.^18^

**Table 2:** GWP_bio_ factors for different scenarios with a 100-year time horizon.

Rotation: # of years	GWP_bio_		Holtsmark^16^	Cherubini *et al*.^10^	Guest *et al*.^15^
	*η*l = 0%	*η*_*l*_ = 100%.			
30	0.18	0.13	—	0.18	—
50	0.24	0.16	—	0.30	—
100	0.32	0.21	1.25	0.60	0.58

**Table 3:** Biogenic CO_2_ emission from all processes.

Technology	Percentage of each process						Total (kg CO_2_ eq)
	Feedstock Collection	Transportation, Storage and Preprocessing	Thermochemical conversion	Distribution	Final Usage	Waste Disposal	
BTE	0.00	0.01	65.72	0.01	34.25	0.02	289
BLFP	0.00	0.01	47.42	0.01	52.55	0.01	144.6
CBTL	0.00	0.27	19.97	0.01	80.17	0.00	19.8
Power	0.00	0.03	99.84	0.11	0.00	0.02	235.38
Pellet	0.00	0.19	2.05	0.01	97.74	0.01	57.62

## References

[b1] Ragauskas A. J. . The Path Forward for Biofuels and Biomaterials. Sci. 311 **(5760)**, 484–489 (2006). 10.1126/science.1114736 16439654

[b2] Zeman F. S. & Keith D. W. Carbon neutral hydrocarbons. Philosophical Transactions of the Royal Society of London A: Mathematical, Physical and Engineering Sciences, 366(**1882)**, 3901–3918 (2008). 10.1098/rsta.2008.0143 18757281

[b3] *Estimation of greenhouse gas emissions and sinks*; Final report from the OECD experts meeting; Organization for Economic Cooperation and Development: Pairs, France (1991).

[b4] Gytarsky M. . *IPCC Guidelines for national greenhouse gas inventories*, Prepared by the National Greenhouse Gas Inventories Program. Intergovernmental Panel on Climate Change: Geneva, Switzerland, http://www.ipcc-nggip.iges.or.jp/public/2006gl (2006) (Date of access: 10/05/2016).

[b5] Frischknecht R. J. . *Implementation of Life Cycle Impact Assessment Methods: Data v2.0. ecoinvent report No. 3*; Swiss center for Life Cycle Inventories: Zurich, Switzerland (2007).

[b6] Foster B. *IPCC Third Assessment Report. The Scientific Basis. Intergovernmental Panel on Climate Change (IPCC)*; Intergovernmental Panel on Climate Change: Geneva, Switzerland, https://www.ipcc.ch/ipccreports/tar/wg1 (2001) (Date of access:01/05/2016).

[b7] Pachauri R. K. & Reisinger A. *IPCC Fourth Assessment Report. The Physical Science Basis. Intergovernmental Panel on Climate Change (IPCC)*.Intergovernmental Panel on Climate Change: Geneva, Switzerland, https://www.ipcc.ch/publications_and_data/publications_ipcc_fourth_assessment_report_wg1_report_the_physical_science_basis.htm (2007) (Date of access: 02/05/2016).

[b8] Cherubini F. & Strømman A. H. Life cycle assessment of bioenergy systems: State of the art and future challenges. Bioresource Technol. 102 **(2)**, 437–451 (2011). 10.1016/j.biortech.2010.08.010 20832298

[b9] Shonnard D. R. . A Review of Environmental Life Cycle Assessments of Liquid Transportation Biofuels in the Pan American Region. Environ. Manage. 56 **(6)**, 1356–1376 (2015). 26041501 10.1007/s00267-015-0543-8

[b10] Cherubini F., Peters G. P., Berntsen T., Strømman A. H. & Hertwich E. CO_2_ emissions from biomass combustion for bioenergy: atmospheric decay and contribution to global warming. GCB Bioenergy. 3 **(5)**, 413–426 (2011).

[b11] Johnson E. Goodbye to carbon neutral: Getting biomass footprints right. Environ. Impact Asses. 29 **(3)**, 165–168 (2009).

[b12] Searchinger T. D. . Fixing a Critical Climate Accounting Error. Sci. 326 **(5952)**, 527–528 (2009). 10.1126/science.1178797 19900885

[b13] Pingoud K., Ekholm T. & Savolainen I. Global warming potential factors and warming payback time as climate indicators of forest biomass use. itig. Adapt. Strategies Glob. Chang. 17 **(4)**, 369–386 (2012).

[b14] Bright R. M., Cherubini F. & Stromman A. H. Climate impacts of bioenergy: Inclusion of carbon cycle and albedo dynamics in life cycle impact assessment. Environ. Impact Asses. 37, 2–11 (2012).

[b15] Guest G., Cherubini F. & Stromman A. H. The role of forest residues in the accounting for the global warming potential of bioenergy. GCB Bioenergy. 5 **(4)**, 459–466 (2013).

[b16] Holtsmark B. Quantifying the global warming potential of CO_2_ emissions from wood fuels. GCB Bioenergy. 7 **(2)**, 195–206 (2015).

[b17] Holtsmark B. A comparison of the global warming effects of wood fuels and fossil fuels taking albedo into account. GCB Bioenergy. 7 **(5)**, 984–997 (2015).

[b18] Asante P., Armstrong G. W. & Adamowicz W. L. Carbon sequestration and the optimal forest harvest decision: A dynamic programming approach considering biomass and dead organic matter. Journal of Forest Economics 17 **(1)**, 3–17 (2011).

[b19] Løken Ø., Eriksen R., Astrup R. & Eid T. *The total biomass of trees in Norway*. Norwegian Institute for Forest and Landscape: Ås, Norway (2012).

[b20] Tuomi M., Laiho R., Repo A. & Liski J. Wood decomposition model for boreal forests. Ecol. Model. 222, 709–718 (2010).

[b21] Joos F. . Carbon dioxide and climate implse response functions for the computation of greenhouse gas metrics: a multi-model analysis. Atmos. Chem. Phys. 13, 2793–2825 (2013).

[b22] Myhre G. . Anthropogenic and natural radiative forcing. In: Climate change 2013: The physical science basis. Contribution of working group I to the fifth assessment report of the Intergovernmental Panel on Climate Change. Stocker T. F., Qin D., Plattner G.-K., Tignor M., Allen S. K., Boschung J., Nauels A., Xia Y., Bex V., Midgley P. M.Cambridge University Press, Cambridge, United Kingdom and New York, NY, USA (2012).

[b23] Keesom W., Unnasch S., Moretta J. & Consultancy J. *Life cycle assessment comparison of North American and imported crudes*. Alberta Energy Research Institute: Alberta, Canada, http://www.eipa.alberta.ca/media/39640/life%20cycle%20analysis%20jacobs%20final%20report.pdf (2009) (Date of access: 15/04/2016).

[b24] Liu W. Economic and Environmental Analyses of Biomass Utilization for Bioenergy Products in the Northeastern United States. Diss. West Virginia University (2015).

[b25] Mckechnie J., Colombo S., Chen J., Mabee W. & Maclean H. L. Forest bioenergy or forest carbon? Assessing trade-offs in greenhouse gas mitigation with wood-based fuels. Environ. Sci. Technol. 45, 789–795 (2011). 21142063 10.1021/es1024004

[b26] Koellner T. . UNEP-SETAC guideline on global land use impact assessment on biodiversity and ecosystem services in LCA. Int. J. Life Cycle Assess. 18, 1188–1202 (2013).

[b27] Gadow K. v. & Hui G. Modeling forest development. Kluwer Academic Publishers, Dordrecht: 212 p (1999).

[b28] Merino A., Balboa M. A., Soalleiro R. R. & Álvarez González J. G. Nutrient exports under different harvesting regimes in fast-growing forest plantations in southern Europe. Forest Ecol. Manag. 207 **(3)**, 325–339 (2005).

[b29] Khanna M., Dhungana B. &Clifton-Brown J. Costs of producing miscanthus and switchgrass for bioenergy in Illinois. Biomass Bioenerg. 32 **(6)**, 482–493 (2008).

[b30] Dale V. H. . Climate change and forest disturbances. Bioscience. 51 **(9)**, 723–734 (2001).

[b31] Westerling A. L., Hidalgo H. G., Cayan D. R. & Swetnam T. W. Warming and earlier spring increase western US forest wildfire activity. Sci. 313 **(5789)**, 940–943 (2006). 10.1126/science.1128834 16825536

[b32] Colwell R. K., Brehm G., Cardelus C. L., Gilman A. C. & Longino J. T. Global warming, elevational range shifts, and lowland biotic attrition in the wet tropics. Sci. 322 **(5899)**, 258–261 (2008). 10.1126/science.1162547 18845754

[b33] Boisvenue C. & Running S. W. Impacts of climate change on natural forest productivity - evidence since the middle of the 20th century. Glob. Change Biol. 12 **(5)**, 862–882 (2006).

[b34] Fantozzi F. & Buratti C. Life cycle assessment of biomass chains: Wood pellet from short rotation coppice using data measured on a real plant. Biomass Bioenergy. 34 **(12)**, 1796–1804 (2010).

[b35] Hsu D. D. . Life cycle environmental impacts of selected US ethanol production and use pathways in 2022. Environ. Sci. Technol. 44 **(13)**, 5289–5297 (2010). 20527764 10.1021/es100186h

[b36] Hsu D. D. Life cycle assessment of gasoline and diesel produced via fast pyrolysis and hydroprocessing. Biomass Bioenergy. 45, 41–47 (2012).

[b37] Kumar D. & Murthy G. S. Life cycle assessment of energy and GHG emissions during ethanol production from grass straws using various pretreatment processes. Int. J. Life Cycle Assess. 17 **(4)**, 388–401 (2012).

[b38] Snowden-Swan L. J. & Male J. L. *Summary of Fast Pyrolysis and Upgrading GHG Analyses*. Richland, WA, http://www.pnnl.gov/main/publications/external/technical_reports/PNNL-22175.pdf (2012) (Date of access: 16/04/2016).

[b39] Tarka T. J. . Affordable, low-carbon diesel fuel from domestic coal and biomass. In Proc. 25th Annual Pittsburgh Coal Conf., Pittsburgh, PA. (2009).

[b40] Spath P. L., Mann M. K. & Kerr D. R. *Life cycle assessment of coal-fired power production*. National Renewable Energy Lab.: Golden, CO. http://www.nrel.gov/docs/fy99osti/25119.pdf (1999) (Date of access: 15/04/2016).

[b41] Yancey N. A., Tumuluru J. S. & Wright C. T. Drying, Grinding and Pelletization Studies on Raw and Formulated Biomass Feedstock’s for Bioenergy Applications. J. Biobased Mate. Bioenergy. 7 **(5)**, 549–558 (2013).

[b42] Environmental Protection Agency. Regulation of Fuels and Fuel Additives: Changes to Renewable Fuel Standard Program; Final Rule, Washington D.C., United States, https://www.gpo.gov/fdsys/pkg/FR-2010-03-26/pdf/2010-3851.pdf (2010) (Date of access: 10/08/2016).

