# Supplementary material for: Analysis of the Global Warming Potential of Biogenic CO2 Emission in Life Cycle Assessments
Source: Sci Rep. 2017 Jan 3;7:39857. doi: 10.1038/srep39857 (PMC5206676; doi:10.1038/srep39857)
Supplement: Supplementary Information [file srep39857-s1.pdf]

Supporting Information for

Analysis of the Global Warming Potential of Biogenic CO_2 Emission in Life Cycle Assessments

Weiguo Liu[1], Zhonghui Zhang[2], Xinfeng Xie[*3], Zhen Yu[4], Klaus von Gadow[5], Junming Xu[6], Shanshan Zhao[2], Yuchun Yang[2]

[1]*School of Natural Resources, West Virginia University, Morgantown, WV 26506, United States.*
[2]*Jilin Province Academy of Forestry Research, Changchun, 130033, China.*
3 School of Forest Resources and Environmental Science, Michigan Technological University, Houghton, MI 49931, United States.
[4]*Department of Ecology, Evolution, and Organismal Biology (EEOB), Iowa State University, Ames, IA 50011, United States.*
[5]*Burckhardt Institute, Georg-August University Göttingen, Göttingen, Germany*
[6] *Institute of Chemical Industry of Forest Products CAF, Nanjing, Jiangsu, China.*

[*]*Corresponding author*

Table of Content

Decomposition of residue 3
Setting of mass allocation 4
Sensitivity Analysis 6
System boundary of LCA case studies 7
Life cycle inventory 9
Change of forest carbon 10

Decomposition of residue

Soil carbon model Yasso07 was used in this study to simulate the decomposition of residues (Tuomi et al. 2009). The parameter values were set by Liski et al. (2009). The decomposition rate of soil carbon inputs depends on chemical compartments of inputs which were also determined by the average of chemical composition of residue types (Liskiet al. 2009). The detailed chemical composition of leaf/needle and branch/stem/root is listed in table S1. Fig. S1 shows the decomposition rate of leaf/needle and branch/stem/root within 100 years.

Table S1. Chemical composition of residue types.

Type	Acid hydrolysables	Water solubles	Ethanol solubles	neither soluble nor hydrolysables
Leaf/needle	45%	24%	9%	22%
Branch/stem/root	52%	2%	6%	40%

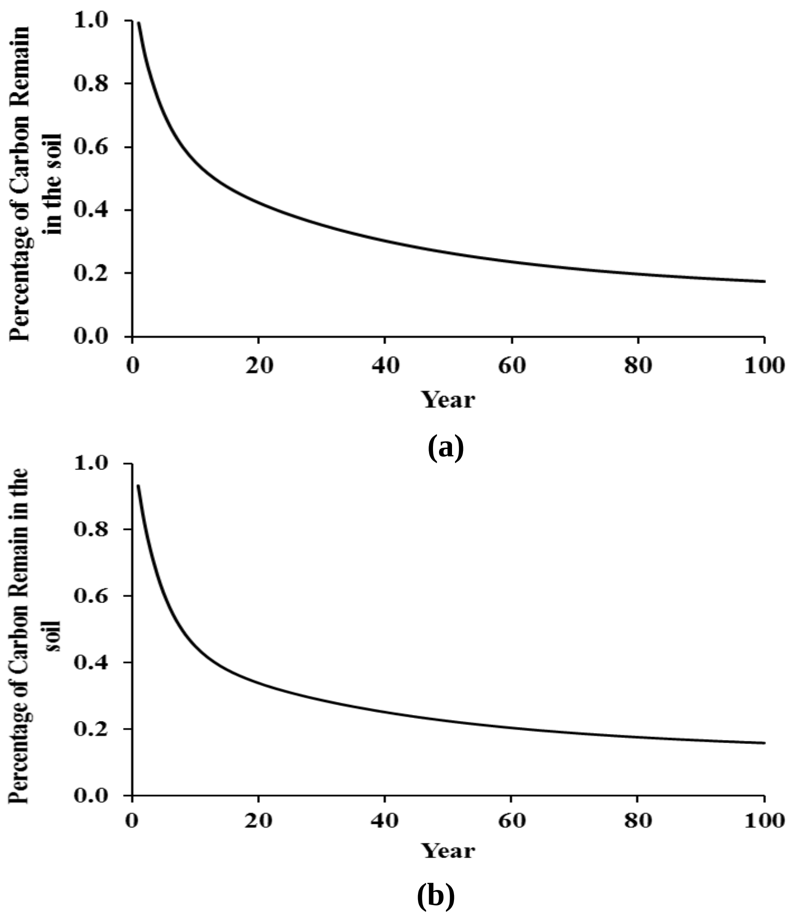

Fig. S1. The decomposition rate of residue simulated by Yasso07: (a) branch/stem/root; (b) leaf/needle.

Setting of mass allocation

The total emissions ($E_{total}(t)$) remain in the atmosphere are from four compartments: biomass combustion ($E_b(t)$), soil carbon decomposition ($E_s(t)$),long-lived woody products ($E_{wpl}(t)$) and short-lived woody products ($E_{wps}(t)$).

$$E_{total}(t) = E_b(t) + E_s(t) + E_{wpl}(t) + E_{wps}(t) \qquad (S-1)$$

The mass allocation coefficient of removed biomass ($\sigma_b(t)$) is calculated based on the CO_2 emissions remained in the atmosphere in each year. Thus,

$$\sigma_b(t) = \frac{E_b(t)}{E_{total}(t)} \qquad (S-2)$$

The decay rate of CO_2 emissions is not following a simple line. The fraction of its initial pulse of CO_2 at time *t* is labeled as *y(t)* which is calculated by the equation (1) in the main manuscript. Thus, $E_b(t)$ is calculated as:

$$E_b(t) = \omega(B-T)\cdot y(t) \qquad (S-3)$$

Where ω is the proportion of residues that are collected for bioenergy.B is the amount of biomass in tC/ha before harvest and T is merchantable timber in tC/ha.

$$E_s(t) = \int_0^t [E_l(t') + E_{nl}(t')]\cdot y(t-t')dt'$$

$$= \int_0^t [p_l\,(B-T)d_l(t') + (1-p_l-\omega)(B-T)d_{nl}(t')]\cdot y(t-t')dt'$$

$$= \int_0^t [p_l\,d_l(t') + (1-p_l-\omega)d_{nl}(t')](B-T)\cdot y(t-t')dt' \qquad (S-4)$$

Where $E_l(t)$ is emission from leaf, and $E_{nl}(t)$ is emission from branch, stem and root. $p_l$is the percentage of leaf in remain biomass after harvest. $d_l(t)$is decomposition rate of leaf, and $d_{nl}(t)$ is decomposition rate of branch, stem and root.

$$E_{wps}(t) = \begin{cases} 0, t < L_s \\ \eta_s T y(t-L_s), t \geq L_s \end{cases} \qquad (S-5)$$

$$E_{wpl}(t) = \begin{cases} 0, t < L_l \\ \eta_l T y(t-L_l), t \geq L_l \end{cases} \qquad (S-6)$$

Where L_s and L_l are the life span of short-lived and long-lived woody products.$\eta_s$andη_l are percentage of short-lived and long lived woody products in the total merchantable timber.

In this study, we assumedp_l, η_s, η_l are 10%, 70% and 30% respectively. Two scenarios were studied: worst case ($\eta_l = 0\%$) and best case ($\eta_l = 100\%$). The mass allocation coefficient ($\sigma_b(t)$) is shown in Fig. S2. Too simplify the model, the life span of life-lived woody products is

set to 100 years to ensure that it is longer than RL, where RL is rotation length. The short-lived woody product will emit CO_2 right after harvest.

When the residue is not removed, the mass allocation coefficient of this amount of residue ($\sigma_d(t)$) is calculated in the same by replacing $E_b(t)$with $E_d(t)$, where $E_d(t)$ is:

$$E_d(t) = \int_0^t \omega(B-T)d_{nl}(t') \cdot y(t-t')dt' \qquad (S-7)$$

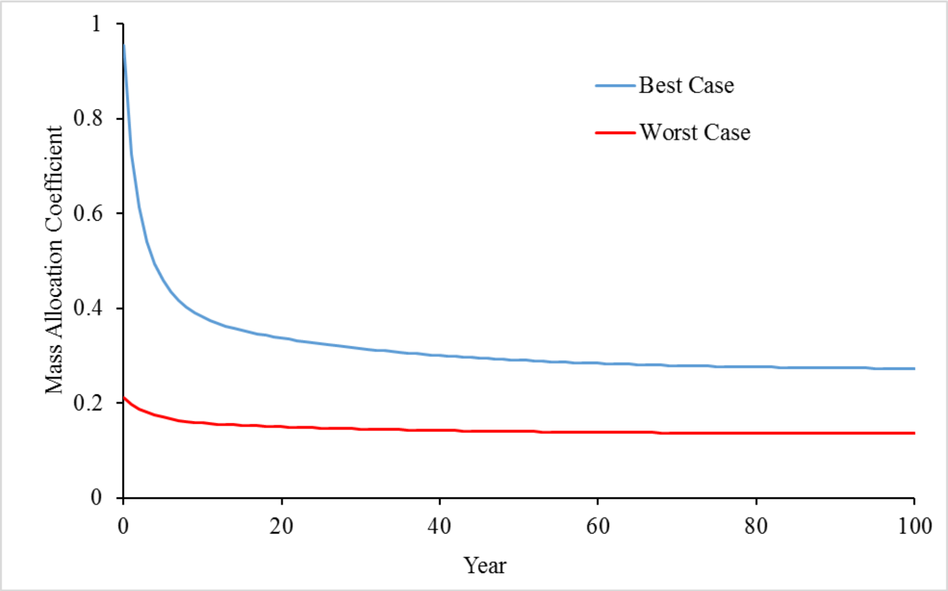

Fig. S2. The mass allocation coefficient in the two scenarios.

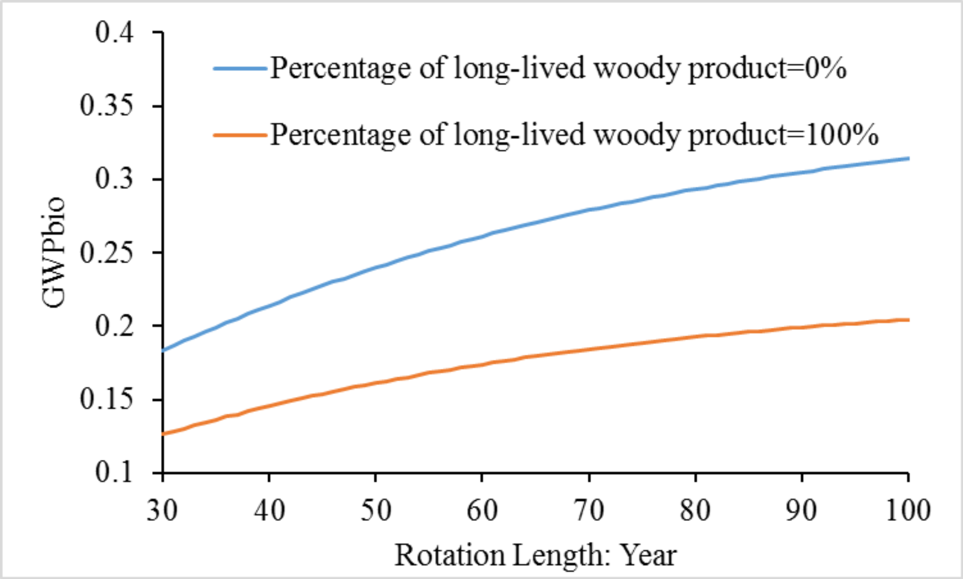

Fig. S3. The sensitivity analysis of rotation length for GWP_{bio}.

Sensitivity Analysis

Two major factors need to be tested for sensitivity: rotation length (RL) and percentage of long-lived woody product (η_l). The sensitivity analysis of rotation length from 30 years to 100 years is shown in Fig. S3. The growth function of forest with different rotation length were obtained by change the setting of the Chapman-Richards growth model. Because there is no way to validate the settings of the parameters, this sensitivity analysis can only be considered as a reasonable approach. The results of sensitivity analysis of η_l showed a linear correlation between $GWP_{bio}$and η_l. Thus, the GWP_{bio} for certain η_l can be easily obtained by two extreme scenarios ($\eta_l = 0\%$and $\eta_l = 100\%$), and no table and figure is provided in the Supplemental Information.

System boundary of LCA case studies

Five cases were included in this study: biomass to ethanol, biomass to liquid fuel via fast pyrolysis, coal and biomass to liquids, biomass to bio-power and biomass to pellet fuel. The cradle to grave assessment include collection, transportation, storage, preprocessing, bio-product conversion, distribution, final usage and waste disposal. The detailed system boundaries are shown in Fig. S4-S7.

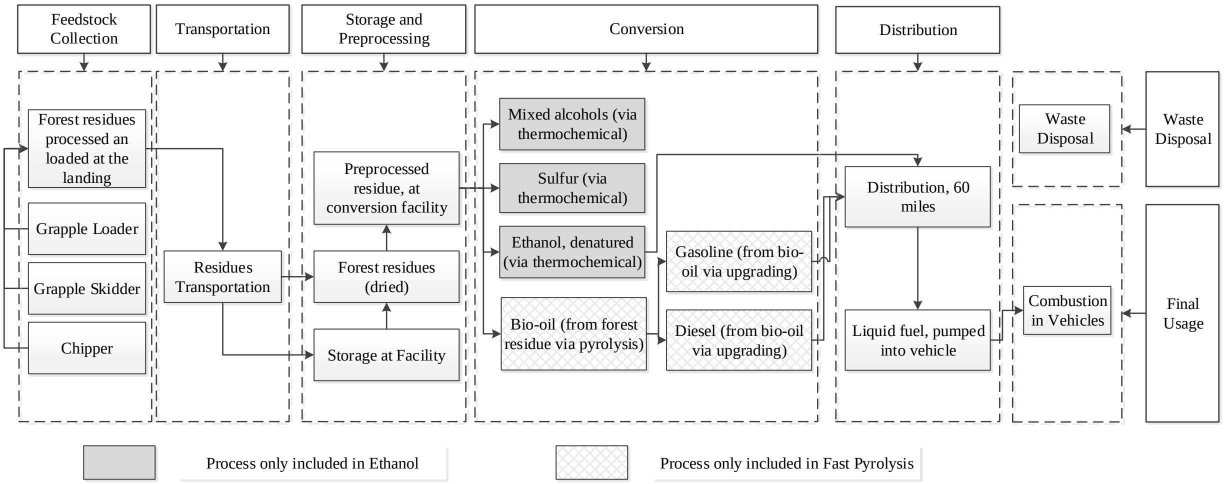

Fig.S4. System boundary of biomass to ethanol and biomass to liquid fuels via fast pyrolysis.

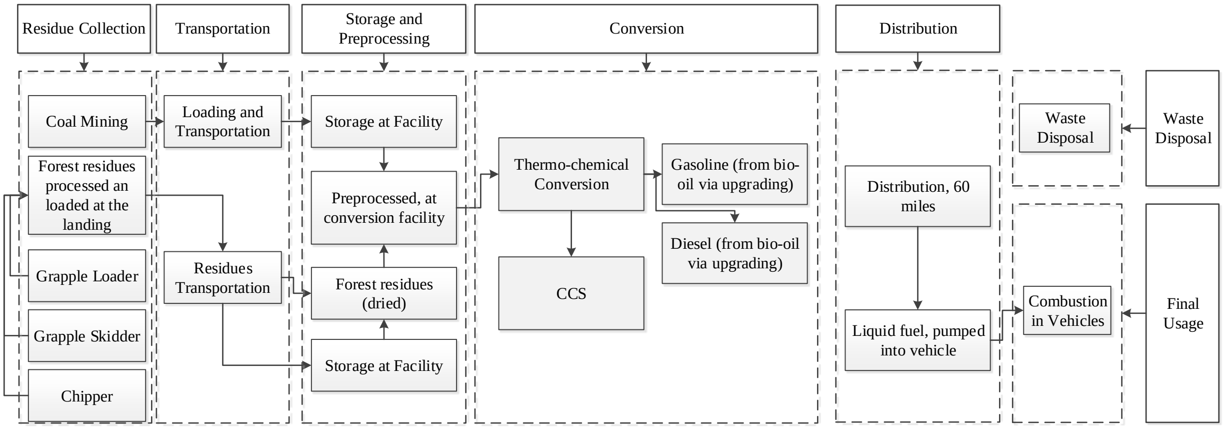

Fig.S5. System boundary of coal and biomass to liquids.

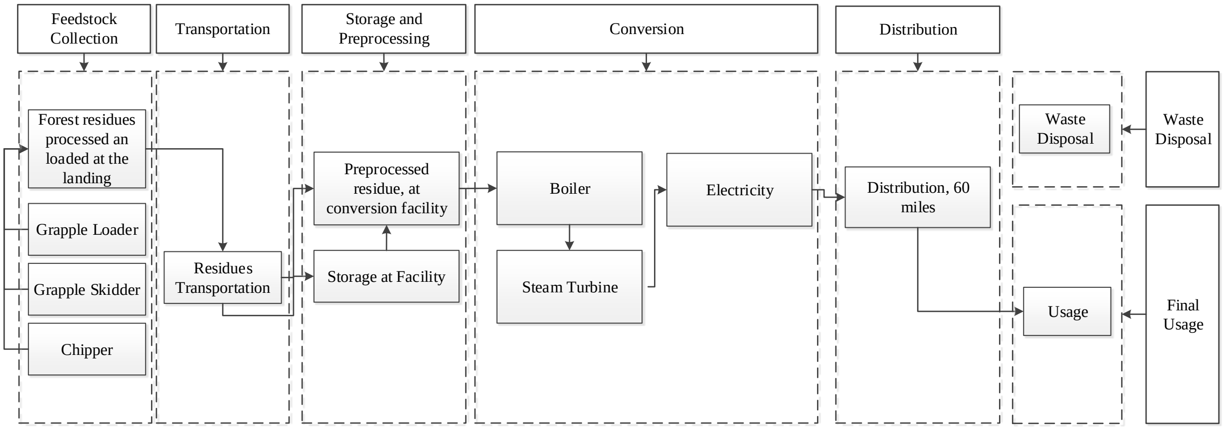

Fig. S6. System boundary of biomass to bio-power.

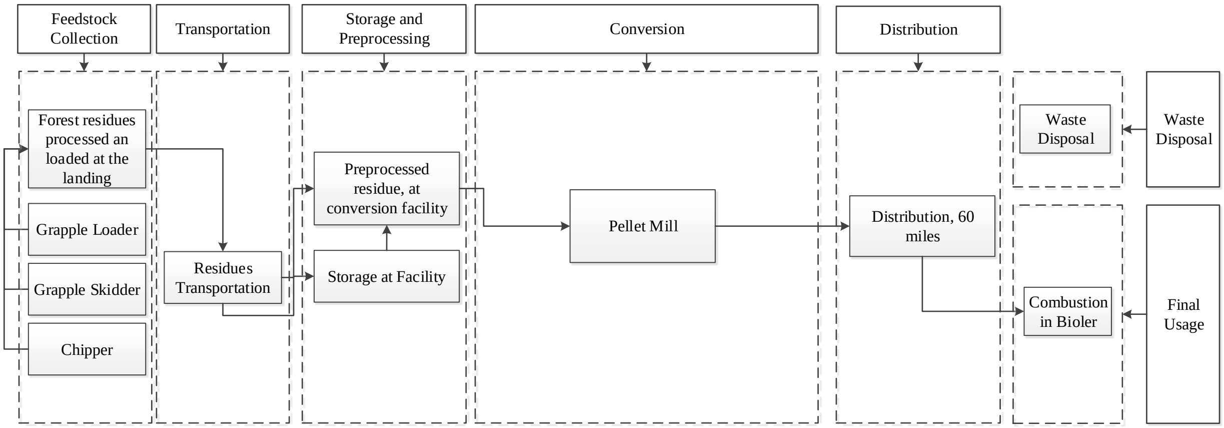

Fig S7. System boundary of biomass to pellet.

Life cycle inventory

The detailed data sources for the inventory data are listed in Table S2.

Table S2. Source of life cycle inventory data.

Processes	Biomass to Ethanol	Biomass to Liquid Fuel via Fast Pyrolysis	Coal and Biomass to Liquids	Biomass to Bio-power	Biomass to Pellet
Feedstock Collection	Wu *et al.* 2012	Wu *et al.* 2012	Wu *et al.* 2012; US LCI.	Wu *et al.* 2012	Wu *et al.* 2012
Transportation	Ecoinvent 3.	Ecoinvent 3.	Ecoinvent 3.	Ecoinvent 3.	Ecoinvent 3.
Storage and Preprocessing	Emery and Mosier 2012; US LCI; Idaho National Laboratory Process Demonstration Unit	Emery and Mosier 2012; US LCI; Idaho National Laboratory Process Demonstration Unit	Emery and Mosier 2012; US LCI; Idaho National Laboratory Process Demonstration Unit	Emery and Mosier 2012; US LCI; Idaho National Laboratory Process Demonstration Unit	Emery and Mosier 2012; US LCI; Idaho National Laboratory Process Demonstration Unit
Conversion	Hsu 2010	Hsu 2012	WVU Chemical Engineering lab simulation	Spath*et al.* 1999	INL PDU
Distribution	Marano and Cifenrno 2001	Marano and Cifenrno 2001	Marano and Cifenrno 2001	Marano and Cifenrno 2001	Marano and Cifenrno 2001
Final Usage	Hsu 2010	Hsu 2012	WVU Chemical Engineering lab simulation	-	Brassard *et al.* 2014
Waste Disposal	Ecoinvent 3.	Ecoinvent 3.	Ecoinvent 3.	Ecoinvent 3.	Ecoinvent 3.

Change of forest carbon

Traditional LCA quantifies the emissions relate to the production of bioproduct and harvest of biofuels. Forest carbon change due to the collection of biomass should be included.

$$FC_b = GWP_d E'_s(t) \cdot \frac{\omega(B-T)}{\omega(B-T)+T} \qquad (S-8)$$

$$E'_s(t) = \int_0^{t^{comp}} [p_l(B-T)d_l(t') + (1-p_l-\omega)(B-T)d_{nl}(t')]dt' \qquad (S-9)$$

Where t^{comp} is the year when the emission from soil organic matter is fully compensated by forest regrowth. GWP_d is calculated as formula (8) in the manuscript. The GWP_d is shown in Table S3. To calculate the LCA emissions, 3.667$GWP_d \cdot FC_b$ should be add to the total LCA GHG emissions. A coefficient 3.667 is multiplied to convert tC to ton CO_2 eq.

Table S3. GWP_d values for different cases.

	GWP_d	
Rotation: years	$\eta_l = 0\%$	$\eta_l = 100\%$
30	0.07	0.04
50	0.10	0.07
100	0.12	0.09

References

Brassard, P.; Palacios, J. H.; Godbout, S.; Bussieres, D.; Lagace, R.; Larouche, J. P.; Pelletier, F., Comparison of the gaseous and particulate matter emissions from the combustion of agricultural and forest biomasses. *Bioresource Technology* **2014,** *155*, 300-306.

Emery, I. R.; Mosier, N. S., The impact of dry matter loss during herbaceous biomass storage on net greenhouse gas emissions from biofuels production. *biomass and bioenergy* **2012,** *39*, 237-246.

Hsu, D.D. 2012. Life cycle assessment of gasoline and diesel produced via fast pyrolysis and hydroprocessing. Biomass and Bioenergy, 45: 41-47. 2009. Finnish Environment Institute, Helsinki, Finland.

Hsu, David D., Inman, Daniel, Heath, Garvin A., Wolfrum, Edward J., Mann, Margaret K., Aden, Andy. 2010. Life cycle environmental impacts of selected U.S. ethanol production and use pathways in 2022. Environmental Science and Technology, 44: 5289-5297.

Liski, J., Tuomin, M., Rasinmäki, J. Yasso07 user-interface manual. **2009**. Finnish Environment Institute, Helsinki, Finland. http://www.syke.fi/download/noname/%7B86C64459-9FFF-4AB9-8DA0-026BE5652F48%7D/39582.

Marano, J.J.; Ciferno, J.P. Life-cycle Greenhouse-Gas Emissions Inventory for Fischer– Tropsch Fuels. Prepared for US Department of Energy National Energy Technology Laboratory. Energy and Environmental Solutions, LLC.: Anaheim, CA, US, 2001.

Spath, P. L.; Mann, M. K.; Kerr, D. R. *Life cycle assessment of coal-fired power production*; National Renewable Energy Lab., Golden, CO (US): Golden, CO, U.S., 1999.

Tuomi, M., Thum, T., Järvinen, H., Fronzek, S., Berg, B., Harmon, M., Trofymow, J.A., Sevanto, S., Liski, J. Leaf litter decomposition – estimates of global variability based on Yasso07 model. *Ecol. Model*.**2009**, *220*,3362-3371.

Wu, J.; Wang,J.; Cheng, Q.; DeVallance, D.B. Assessment of coal and biomass to liquid fuels in central Appalachia, USA. *International Journal of energy Research*. 2012,36, 856-870.
