# Supplementary material for: Analysis of the Global Warming Potential of Biogenic CO2 Emission in Life Cycle Assessments
Source: Sci Rep. 2017 Jan 3;7:39857. doi: 10.1038/srep39857 (PMC5206676; doi:10.1038/srep39857)
Supplement: Supplemental Information [file srep39857-s2.doc]

**Inventory Dataset for**

**Analysis of the Global Warming Potential of Biogenic CO_2_ Emission in Life Cycle Assessments**

Weiguo Liu^1^, Zhonghui zhang^2^, Xinfeng Xie^*3^, Zhen Yu^4^, Klaus von Gadow^5^, Junming Xu^6^, Shanshan Zhao^2^, Yuchun Yang^2^

^1^*School of Natural Resources, West Virginia University, Morgantown, WV 26506, United States.* ^2^*Jilin Province Academy of Forestry Research, Changchun, 130033, China.*

*3 School of Forest Resources and Environmental Science, Michigan Technological University, Houghton, MI 49931, United States.*

*^4^Department of Ecology, Evolution, and Organismal Biology (EEOB), Iowa State University, Ames, IA 50011, United States.*

*^5^Burckhardt Institute, Georg-August University Göttingen, Göttingen, Germany*

^6^ *Institute of Chemical Industry of Forest Products CAF, Nanjing, Jiangsu, China.*

*^*^Corresponding author*

## Life cycle inventory data

| *Process Name* | *Table Number* |
| --- | --- |
| Loaded and transported to Prep Plant | 2 |
| Coal (dried, stored) | 3 |
| Grinding (Coal) | 4 |
| Preprocessed coal, at conversion facility | 5 |
| Grapple Skidder | 6 |
| Grapple Loader | 7 |
| Chipper | 8 |
| Forest residues processed and loaded at the landing | 9 |
| Forest residue (dried, stored) | 10 |
| Preprocessed residue, at conversion facility | 11 |
| CBTL (Syngas) | 12 |
| CBTL (Diesel) | 13 |
| Distribution, 60 miles | 14 |
| Liquid fuels pumped into vehicle | 15 |
| Transmission of Electricity | 16 |
| Gasoline Combustion | 17 |
| Diesel Combustion | 18 |
| Thermochemical conversion plant | 19 |
| Indirect heated softwood | 20 |
| Dry wood residue combustion | 21 |
| Residue Dried | 22 |
| Denatured ethanol | 23 |
| Distribution, 60 miles | 24 |
| Ethanol, forest residue, at blending terminal | 25 |
| Liquid fuels pumped into vehicle | 26 |
| Ethanol combustion | 27 |
| Bio-oil | 28 |
| Upgrade | 29 |
| Gasoline combustion | 30 |
| Diesel combustion | 31 |
| Wheel Loader L150G | 32 |
| Active Drier, MC<10%, Forest residue | 33 |
| Grinder, Particle size<2mm, Forest residue | 34 |
| Hammer Mill, Particle size<2mm, Forest residue | 35 |
| Preprocess, Pyrolysis, Forest residue | 36 |
| Grinder, Particle size<1/4", Forest residue | 37 |
| Hammer Mill, Particle size<1/4", Forest residue | 38 |
| Preprocess, Pellet | 39 |
| Cooling | 40 |
| Power Plant, Biomass | 41 |
| Pellet Mill, Forest residue | 42 |
| Pellet, distribution | 43 |
| Pellet, combustion, Forest residue | 44 |
| Feller-Buncher | 45 |

*Table 1 Processes involved in on the LCA models.*

*Table 2*. Process “Loaded and transported to Prep Plant”.

| **Products and co-product** | |
| --- | --- |
| Loaded and transported to Prep Plant | *1 ton* |
| ***Materials/fuels*** | |
| Transport, lorry 16-32t, EURO5/RER U^a^ | *8 tkm* |
| Bituminous Coal, at mine^b^ | *1 ton* |

^a^Ecoinvent 2.2;

^b^ US-LCI.

*Table 3*. Process “Coal (dried, stored)”.

| **Products and co-product** | |
| --- | --- |
| Coal (dried, stored)^a^ | 0.98 *ton* |
| **Materials/fuels** | |
| Loaded and transported to Prep Plant | 1 *ton* |
| Transport, freight, rail, diesel/US U^b^ | 29.68 *tkm* |
| Fodder loading, by self-loading trailer/CH with US electricity US | 2.27 m^3^ |

^a^ Assuming 2% dry coal loss;

^b^Ecoinvent 2.2.

*Table 4*. Process “Grinding (Coal)”.

| **Products and co-product** | |
| --- | --- |
| Grinding (Coal)^a^ | 2 *ton* |
| **Materials/fuels** | |
| Electricity, at Grid, US, 2008/RNA U^b^ | 6.19E1 *kWh* |

^a^ Revised from US-LCI;

bEcoinvent 2.2.

*Table 5*. Process “Preprocessed coal, at conversion facility”.

| **Products and co-product** | |
| --- | --- |
| Preprocessed coal, at conversion facility | 1 *ton* |
| **Materials/fuels** | |
| Grinding (Coal) | 1 *ton* |
| Coal (dried, stored) | 1 *ton* |

*Table 6*. Process “Grapple Skidder”.

| **Products and co-product** | |
| --- | --- |
| Grapple Skidder^a^ | 24 *ton* |
| **Materials/fuels** | |
| Diesel, combusted in industrial equipment/US^b^ | 13.758 *gal* |
| Lubricant oil (1)^b^ | 0.247644 *gal* |

^a^ Wu, Jinzhuo, Wang, Jingxin, Cheng, Qingzheng, DeVallance, David. 2011. Assessment of coal and biomass to liquid fuels in central Appalachia, USA. International Journal of Energy Research. 36(7): 856-870;

^b^Ecoinvent 2.2.

*Table 7*. Process “Grapple Loader”.

| **Products and co-product** | |
| --- | --- |
| Grapple Loader^a^ | 24 *ton* |
| **Materials/fuels** | |
| Diesel, combusted in industrial equipment/US^b^ | 6.54 *gal* |
| Lubricant oil (1)^b^ | 0.1172 *gal* |

^a^ Wu *et al.* 2011;

^b^Ecoinvent 2.2.

*Table 8*. Process “Chipper”.

| **Products and co-product** | |
| --- | --- |
| Chipper^a^ | 24 *ton* |
| **Materials/fuels** | |
| Diesel, combusted in industrial equipment/US^b^ | 14.52 *gal* |
| Lubricant oil (1)^b^ | 0.26136 *gal* |

^a^ Wu *et al.* 2011;

^b^Ecoinvent 2.2.

*Table 9*. Process “Forest residues processed and loaded at the landing”.

| **Products and co-product** | |
| --- | --- |
| Forest residues processed and loaded at the landing^a^ | 1 *ton* |
| **Natural Resources** | |
| Carbon dioxide, in air | 942 *kg* |
| Energy, from biomass | 8561 *MJ* |
| **Materials/fuels** | |
| Grapple Skidder | 1 *ton* |
| Grapple Loader | 1 *ton* |
| Chipper | 1 *ton* |

^a^ Revised from “Hsu, David D., Inman, Daniel, Heath, Garvin A., Wolfrum, Edward J., Mann, Margaret K., Aden, Andy. 2010. Life cycle environmental impact of selected U.S. ethanol production and use pathway in 2022. Environmental Science and Technology. 44: 5289-5297”;

*Table 10*. Process “Forest residues (dried, stored)”.

| **Products and co-product** | |
| --- | --- |
| Forest residue (dried, stored)^a^ | 0.772 *ton* |
| **Materials/fuels** | |
| Forest residues processed and loaded at the landing | 0.62 *ton* |
| Transport, lorry 16-32t, EURO5/RER U^b^ | 148.73 *tkm* |
| Dried roughage store, non ventilated/CH/I U^b^ | 9.75E-8 *m^3^* |
| Conveyor belt, at plant/RER/I U^b^ | 3.47E-5 *m* |
| Fodder loading, by self-loading trailer/CH with US electricity US | 2.27 m^3^ |
| Sawmill Residue | 0.16 *ton* |

^a^ Revised from “Hsu *et al.* 2010”;

^b^Ecoinvent 2.2.

*Table 11*. Process “Preprocessed residue, at conversion facility”.

| **Products and co-product** | |
| --- | --- |
| Preprocessed residue, at conversion facility^a^ | 1 *ton* |
| **Materials/fuels** | |
| Forest residue (dried, stored) | 1 *ton* |
| Transport, lorry 16-32t, EURO5/RER U^b^ | 20 *tkm* |

^a^ Revised from “Hsu *et al.* 2010”;

^b^Ecoinvent 2.2.

*Table 12*. Thermal-conversion Process “CBTL (Syngas)”.

| **Products and co-product** | |
| --- | --- |
| Syncrude^a^ | 165.41 kg |
| Light Gases^a^ | 24.81 kg |
| **Natural Resources** | |
| Water, unspecified natural origin/kg^b^ | 183.85 kg |
| **Materials/fuels** | |
| Preprocessed coal, at conversion facility | 500 kg |
| Preprocessed residue, at conversion facility | 43.3 kg |
| Thermochemical conversion plant^b^ | 5.95E-9 p |
| **Emissions to air** | |
| Carbon dioxide, fossil | 41.5 kg |
| Carbon dioxide, biogenic | 23.3 kg |

^a^ Simulation based on Aspen Plus: Jiang, Yuan, Bhattacharyya, Debangsu. 2015. Modeling and Analysis of an Indirect Coal Biomass to Liquids Plant Integrated with a Combined Cycle Plant and CO_2_ Capture and Storage. Energy and Fuels, 29 (8): 5434-5451.

^b^Ecoinvent 2.2.

*Table 13*. Thermal-conversion Process “CBTL (Diesel)”.

| **Products and co-product** | |
| --- | --- |
| CBTL (Diesel)^a^ | 88.067 kg |
| CBTL (Gasoline)^a^ | 52.966 kg |
| Electricity_CBTL | 122.54 MJ |
| **Natural Resources** | |
| Water, unspecified natural origin/kg^b^ | 65.83 kg |
| **Materials/fuels** | |
| Syncrude^a^ | 165.41 kg |
| Light Gases^a^ | 24.81 kg |
| **Emissions to air** | |
| Carbon dioxide, fossil | 26.9 kg |
| Carbon monoxide, fossil | 1.51 kg |

^a^ Simulation based on Aspen Plus: Jiang, Yuan, Bhattacharyya, Debangsu. 2015. Modeling and Analysis of an Indirect Coal Biomass to Liquids Plant Integrated with a Combined Cycle Plant and CO_2_ Capture and Storage. Energy and Fuels, 29 (8): 5434-5451.

^b^Ecoinvent 2.2.

*Table 14*. Process “Distribution, 60 miles”.

| **Products and co-product** | |
| --- | --- |
| Distribution, 60 miles^a^ | 1 *gal* |
| **Emissions to air** | |
| Carbon dioxide, fossil | 28.29 *g* |
| Methane | 0.0015 *g* |
| Dinitrogen monoxide | 0.0009 *g* |
| Sulfur oxides | 0.1389 *g* |
| Nitrogen oxides | 0.1223 *g* |
| Carbon monoxide, fossil | 0.1638 *g* |
| VOC, volatile organic compounds | 0.0011 *g* |
| Particulates, unspecified | 0.0235 *g* |

^a^ Revised from “Marano and Ciferno 2001”.

*Table 15*. Process “Liquid fuels pumped into vehicle”.

| **Products and co-product** | |
| --- | --- |
| Liquid fuels pumped into vehicle^a^ | 0.2973 *gal* |
| **Electricity/heat** | |
| Electricity, low voltage, at grid/US U^b^ | 0.0026495 *kWh* |
| Liquid storage tank, chemicals, organics/CH/I U^b^ | 9.4e-12 *p* |
| Distribution, 60 miles | 0.297348 gal |
| Rubber and plastics hose and belting | 7.49E-12 USD |
| Measuring and dispensing pumps | 9.17E-15 USD |

^a^ Revised from “Hsu *et al.* 2010”;

^b^Ecoinvent 2.2.

*Table 16*. Process “Transmission of Electricity”.

| **Products and co-product** | |
| --- | --- |
| Electricity, Transmission and distribution^a^ | 1,000 MJ |
| **Electricity/heat** | |
| Zinc, primary, at regional storage/RER with US electricity U | 0.000267 kg |
| Glass tube plant/DE/I with US electricity U | 2.26E+08 p |
| Cement, unspecified, at plant/CH with US electricity U | 4.17E-06 kg |
| Steel | 1.37E-06 kg |
| Electricity_CBTL | 1.00E+03 MJ |

^a^ Revised from Jorge, R.S., Hawkins, T.R., Hertwich, E.G. 2011. Life cycle assessment of electricity transmission and distribution power lines and cables. International Journal of Life Cycle Assessment, 17 (1): 9-15.

*Table 17*. Process “Gasoline Combustion”.

| **Products and co-product** | |
| --- | --- |
| Gasoline Combustion^a^ | 52.966 kg |
| **Electricity/heat** | |
| CBTL (Gasoline) | 52.966 kg |
| Liquid fuels pumped into vehicle | 2.12E+01 |
| **Emissions to air** |  |
| Carbon dioxide, fossil | 1.56E+02 kg |
| Carbon dioxide, biogenic | 8.78E+00 kg |
| Carbon monoxide, fossil | 2.35E+00 kg |
| Nitrogen oxides | 7.41E-02 kg |
| Sulfur oxides | 2.76E-03 kg |
| Methane | 4.27E-03 kg |

^a^ Revised from “Marano and Ciferno 2001”.

*Table 18*. Process “Diesel Combustion”.

| **Products and co-product** | |
| --- | --- |
| Diesel Combustion^a^ | 88.067 kg |
| **Electricity/heat** | |
| CBTL (Diesel) | 88.067 kg |
| Liquid fuels pumped into vehicle | 2.12E+01 |
| **Emissions to air** |  |
| Carbon dioxide, fossil | 2.55E+02 kg |
| Carbon dioxide, biogenic | 1.43E+01 kg |
| Carbon monoxide, fossil | 6.23E-01 kg |
| Nitrogen oxides | 1.42E-01 kg |
| Methane | 4.27E-03 kg |

^a^ Revised from “Marano and Ciferno 2001”.

*Table 19. Process “Thermochemical conversion plant”.*

| **Products and co-product** | |
| --- | --- |
| Thermochemical conversion plant^a^ | 1 p |
| **Materials/fuels** | |
| Concrete, sole plate and foundation, at plant/CH U | 39100 m^3^ |
| Steel, low-alloyed, at plant/RER U | 526000 kg |
| Steel, converter, unalloyed, at plant/RER U | 1240000 kg |
| Chromium steel 18/8, at plant/RER U | 456000 kg |
| Zinc, primary, at regional storage/RER U | 271000 kg |
| Copper, at regional storage/RER U | 113000 kg |
| Nickel, 99.5%, at plant/GLO U | 10100 kg |
| Transport, lorry 20-28t, fleet average/CH U | 3140000 kg |
| Transport, freight, rail/CH U | 1570000 tkm |
| Diesel, burned in building machine/GLO U | 3.84E+05 MJ |
| Electricity, medium voltage, at grid/US U | 4.65E+04 kWh |
| **Emissions to air** | |
| Heat, waste | 1.67E+05 MJ |
| **Waste Treatment** | |
| Disposal, building, concrete gravel, to final disposal/CH S | 8.59E+07 MJ |

^a^ Revised from “Hsu *et al.* 2010”;

*Table 20. Process “Indirect heated softwood”.*

| **Products and co-product** | |
| --- | --- |
| Indirect heated softwood, plywood drying^a^ | 411 kg |
| **Materials/fuels** | |
| Particulates, unspecified | 0.159 kg |
| Carbon monoxide, biogenic | 1.27E-02 kg |

^a^ Revised from “Hsu *et al.* 2010”;

*Table 21. Process “Dry wood residue combustion”.*

| **Products and co-product** | |
| --- | --- |
| Dry wood residue combustion^a^ | 1055 MJ |
| **Emissions to air** | |
| Particulates | 45.5 g |
| Particulates, < 10 um | 33.6 g |
| Particulates, < 2.5 um | 29.5 g |
| Nitrogen oxides | 222 g |
| Sulfur dioxide | 11.4 g |
| Carbon monoxide, biogenic | 272 g |
| Hydrogen chloride | 8.63 g |
| Methane, biogenic | 9.53E+00 g |
| Organic substances, unspecified | 1.77E+01 g |
| VOC, volatile organic compounds | 7.72E+00 g |
| Nitrous acid | 5.90E+00 g |

^a^ Revised from “Hsu *et al.* 2010”;

*Table 22. Process “Residue Dried”.*

| **Products and co-product** | |
| --- | --- |
| Forest residue (dried) | 1055 MJ |
| **Materials/fuels** | |
| Dried roughage store, non ventilated/CH/I U | 0.00 m^3^ |
| Sawmill Residue | 0.16 ton |
| Fodder loading, by self-loading trailer/CH with US electricity U | 2.27 m^3^ |
| Conveyor belt, at plant/RER/I with US electricity U | 0.00 m^3^ |
| Forest residues processed and loaded at the landing | 0.62 ton |
| Transport, lorry 16-32t, EURO5/RER U | 148.73 tkm |

*Table 23. Process “Denatured ethanol”.*

| **Products and co-product** | |
| --- | --- |
| Ethanol, denatured, (from forest residues via thermochemical) ^a^ | 21202 kg |
| Mixed alcohols (from thermochemical) | 3791 kg |
| Sulfur (from thermochemical) | 53.6 kg |
| **Resources** | |
| Oxygen, in air | 77634 kg |
| Nitrogen, in air | 253790 kg |
| Water, cooling, unspecified natural origin/kg | 74002 kg |
| Water, process, unspecified natural origin/kg | 13348 kg |
| **Materials/fuels** | |
| Silica sand, at plant/DE U | 244 kg |
| Thermochemical conversion plant | 5.95E-06 p |
| Magnesium oxide, at plant/RER U | 3.16 kg |
| Zeolite, powder, at plant/RER S | 45.4 kg |
| Chemicals inorganic, at plant/GLO U | 4.63E+01 kg |
| Monoethanolamine, at plant/RER U | 2.72E+01 kg |
| Hydrochloric acid, 30% in H2O, at plant/RER U | 0.4 kg |
| Sodium hydroxide, 50% in H2O, production mix, at plant/RER U | 0.4 kg |
| Sulphite, at plant/RER U | 4.00E-01 kg |
| Chemicals inorganic, at plant/GLO U | 4.54E-01 kg |
| Diesel, low-sulphur, at regional storage/RER U | 3.13E+01 kg |
| Dry wood residue combustion, EPA AP-42 | 3.90E+05 MJ |
| Indirect heated softwood, plywood drying | 41768 kg |
| Forest residue (dried)_Ethanol | 1.13E+05 kg |
| Petrol, unleaded, at regional storage/RER with US electricity U | 276 kg |
| **Emissions to air** | |
| Ammonia | 0.454 kg |
| Carbon dioxide, biogenic | 107598 kg |
| Nitrogen | 2.64E+05 kg |
| Oxygen | 1.20E+04 kg |
| Water | 6.31E+04 kg |
| Nitrogen dioxide | 8.40E+01 kg |
| Sulfur dioxide | 3.91E+01 kg |
| **Waste treatment** | |
| Disposal, wood ash mixture, pure, 0% water, to sanitary landfill/CH U | 1.10E+03 kg |
| Disposal, inert material, 0% water, to sanitary landfill/CH U | 4.54E+01 kg |
| Treatment, sewage, unpolluted, to wastewater treatment, class 3/CH U | 797 kg |

^a^ Revised from “Hsu *et al.* 2010”;

*Table 24*. Process “Distribution, 60 miles”.

| **Products and co-product** | |
| --- | --- |
| Distribution, 60 miles^a^ | 1 *gal* |
| **Emissions to air** | |
| Carbon dioxide, fossil | 28.29 *g* |
| Methane | 0.0015 *g* |
| Dinitrogen monoxide | 0.0009 *g* |
| Sulfur oxides | 0.1389 *g* |
| Nitrogen oxides | 0.1223 *g* |
| Carbon monoxide, fossil | 0.1638 *g* |
| VOC, volatile organic compounds | 0.0011 *g* |
| Particulates, unspecified | 0.0235 *g* |

^a^ Revised from “Marano and Ciferno 2001”.

*Table 25*. Process “Ethanol, forest residue, at blending terminal”.

| **Products and co-product** | |
| --- | --- |
| Ethanol, forest residue, at blending terminal^a^ | 0.81 kg |
| **Electricity/heat** | |
| Ethanol, denatured, (from forest residues via thermochemical)_Ethanol | 0.81 kg |
| Electricity, medium voltage, at grid/US U | 8.60E-04 kWh |
| Liquid storage tank, chemicals, organics/CH/I U | 8.50E-11 p |

^a^ Revised from “Hsu *et al.* 2010”;

*Table 26*. Process “Liquid fuels pumped into vehicle”.

| **Products and co-product** | |
| --- | --- |
| Liquid fuels pumped into vehicle^a^ | 0.2973 *gal* |
| **Electricity/heat** | |
| Electricity, low voltage, at grid/US U^b^ | 0.0026495 *kWh* |
| Liquid storage tank, chemicals, organics/CH/I U^b^ | 9.4e-12 *p* |
| Distribution, 60 miles | 0.297348 gal |
| Rubber and plastics hose and belting | 7.49E-12 USD |
| Measuring and dispensing pumps | 9.17E-15 USD |
| Eth, forest residue, at blending terminal | 1 kg |

^a^ Revised from “Hsu *et al.* 2010”;

^b^Ecoinvent 2.2.

*Table 27*. Process “Ethanol combustion”.

| **Products and co-product** | |
| --- | --- |
| Ethanol combustion^a^ | 0.080135 kg |
| **Electricity/heat** | |
| Carbon dioxide, biogenic | 2.14E+02 g |
| Methane | 6.80E-03 g |
| Nitrous acid | 7.52E-03 g |

^a^ Revised from “Hsu *et al.* 2010”;

*Table 28. Process “Bio-oil”.*

| **Products and co-product** | |
| --- | --- |
| Bio-oil (from wood via pyrolysis)^a^ | 68038.8 kg |
| **Resources** | |
| Water, process, unspecified natural origin/kg | 6000 lb |
| Air | 350000 lb |
| Water, cooling, unspecified natural origin/kg | 180000 lb |
| Water, unspecified natural origin/kg | 84800 lb |
| **Materials/fuels** | |
| Electricity, medium voltage, at grid/US U | 12000 kWh |
| Hydrochloric acid, 30% in H2O, at plant/RER U | 0.667 lb |
| Sodium hydroxide, 50% in H2O, production mix, at plant/RER U | 0.667 lb |
| Sulphite, at plant/RER U | 0.667 lb |
| Chemicals inorganic, at plant/GLO U | 1 lb |
| Thermochemical conversion plant | 5.95E-06 p |
| Forest residue (dried) | 2.83E+05 lb |
| **Emissions to air** | |
| Oxygen | 24400 lb |
| Nitrogen | 270000 lb |
| Water | 180000 lb |
| Hydrogen | 2.01 lb |
| Carbon dioxide, biogenic | 88100 lb |
| Carbon monoxide, biogenic | 504 lb |
| Water | 1.28E+05 lb |
| Water | 2.01E+04 lb |
| Water | 3.20E+04 lb |
| Water | 1.20E+03 lb |
| **Waste treatment** | |
| Disposal, wood ash mixture, pure, 0% water, to sanitary landfill/CH U | 3.60E+03 lb |
| Treatment, sewage, unpolluted, to wastewater treatment, class 3/CH U | 2.18E+00 m^3^ |

^a^ Revised from “Hsu 2011”;

*Table 29. Process “Upgrade”.*

| **Products and co-product** | |
| --- | --- |
| Gasoline (from bio-oil via upgrading) | 28600 lb |
| Diesel (from bio-oil via upgrading) | 38400 lb |
| **Resources** | |
| Water, cooling, unspecified natural origin/kg | 6070 lb |
| Water, unspecified natural origin/kg | 56400 lb |
| Air | 230000 lb |
| **Materials/fuels** | |
| Natural gas, high pressure, at consumer/RER U | 374000 MJ |
| Zeolite, powder, at plant/RER S | 85 lb |
| Zeolite, powder, at plant/RER S | 0.371 lb |
| Zeolite, powder, at plant/RER S | 3.27 lb |
| Electricity, medium voltage, at grid/US U | 12600 MJ |
| Bio-oil (from wood via pyrolysis) | 68038.8 kg |
| Refinery/RER/I U | 3.30E-06 p |
| **Emissions to air** | |
| Water | 2.90E+04 lb |
| Nitrogen | 1.76E+05 lb |
| Oxygen | 9.74E+03 lb |
| Water | 6.83E+01 lb |
| Hydrogen | 1.23E+02 lb |
| Carbon dioxide, biogenic | 1.75E+03 lb |
| Carbon dioxide, biogenic | 6.71E+02 lb |
| Ethane | 4.02E+02 lb |
| Propane | 3.39E+02 lb |
| Isobutane | 3.01E+02 lb |
| Heptane | 3.76E+02 lb |
| Cyclohexane, propyl- | 7.24E+00 lb |
| Hydrocarbons, aliphatic, alkanes, unspecified | 1.52E+00 lb |
| Hydrocarbons, alkanes, cyclo-, C6 | 2.87E+00 lb |
| Xylene | 1.08E+00 lb |
| Water | 6.07E+03 lb |
| Water | 3.41E+02 lb |
| Water | -3.45E+02 lb |
| Carbon dioxide, biogenic | 8.39E+04 lb |

^a^ Revised from “Hsu 2011”;

*Table 30. Process “Gasoline combustion”.*

| **Products and co-product** | |
| --- | --- |
| Gasoline combustion | 0.112 kg |
| **Materials/fuels** | |
| Gasoline (from bio-oil via upgrading) | 0.112 kg |
| Liquid fuels pumped into vehicle | 0.038638215 gal |
| **Emissions to air** | |
| Carbon dioxide, biogenic | 3.43E+02 g |
| Methane, biogenic | 1.00E-02 g |
| Dinitrogen monoxide | 1.20E-02 g |
| VOC, volatile organic compounds | 1.51E-01 g |
| Carbon monoxide | 3.48E+00 g |
| Nitrogen oxides | 6.90E-02 g |
| Particulates, < 10 um | 2.90E-02 g |
| Particulates, < 2.5 um | 1.40E-02 g |
| Sulfur oxides | 6.00E-03 g |

^a^ Revised from “Hsu 2011”;

*Table 31. Process “Diesel combustion”.*

| **Products and co-product** | |
| --- | --- |
| Diesel combustion | 0.0944 kg |
| **Materials/fuels** | |
| Diesel (from bio-oil via upgrading) | 0.0944 kg |
| Liquid fuels pumped into vehicle | 2.8E-02 gal |
| **Emissions to air** | |
| Carbon dioxide, biogenic | 3.02E+02 g |
| Methane, biogenic | 3.08E-03 g |
| Dinitrogen monoxide | 1.23E-02 g |
| VOC, volatile organic compounds | 6.16E-02 g |
| Carbon monoxide | 5.48E-01 g |
| Nitrogen oxides | 8.22E-02 g |
| Particulates, < 10 um | 3.08E-02 g |
| Particulates, < 2.5 um | 1.54E-02 g |
| Sulfur oxides | 2.05E-03 g |

^a^ Revised from “Hsu 2011”;

*Table 32. Process “Wheel Loader L150G”.*

| **Products and co-product** | |
| --- | --- |
| Wheel Loader L150G^a^ | 270000 ton |
| **Materials/fuels** | |
| Sheet rolling, aluminium/RER U | 266 kg |
| Glass fibre, at plant/RER with US electricity U | 3240 kg |
| Polyethylene, LDPE, granulate, at plant/RER with US electricity U | 102 kg |
| Heavy fuel oil, at regional storage/RER with US electricity U | 2992 kg |
| Paper, woodfree, uncoated, at regional storage/RER with US electricity U | 246 kg |
| Wire drawing, steel/RER with US electricity U | 1800 kg |
| Synthetic rubber, at plant/RER with US electricity U | 6960 kg |
| Crude oil, at production/NG with US electricity U | 450491 kg |
| Hard coal, at regional storage/RNA with US electricity U | 5545.23 kg |
| Lignite coal, combusted in industrial boiler NREL /US | 5733 kg |
| Natural gas, production mix, at service station/CH U | 44743 kg |
| Peat, at mine/NORDEL with US electricity U | 33 kg |

^a^ Salman, O., Chen, Y. 2013. Comparative environmental analysis of conventional and hybrid wheel loader technologies. Master of Science Thesis, Stockholm.

*Table 33. Process “Active Drier, MC<10%, Forest residue”.*

| **Products and co-product** | |
| --- | --- |
| Active Drier, MC<10%, Forest residue^a,b^ | 2.865 ton |
| **Materials/fuels** | |
| Electricity, medium voltage, at grid/US with US electricity U | 350 kWh |
| Transport, truck | 208.3636 tkm |
| Plant site storage | 0.289394 ton |

^a^Nordhagen, E. 2011. Drying of wood chips with surplus heat from two hydroelectric plants in Norway. FORMEC, Austria.

^b^ INL PDU.

*Table 34. Process “Grinder, Particle size<2mm, Forest residue”.*

| **Products and co-product** | |
| --- | --- |
| Grinder, Particle size<2mm, Forest residue ^a^ | 1 ton |
| **Materials/fuels** | |
| Conveyor belt, at plant/RER/I with US electricity U | 3.47E-05 m |
| Transport, truck | 72 tkm |
| Plant site storage | 0.1 ton |
| Electricity, medium voltage, at grid/US with US electricity U | 45.89 kWh |

^a^ INL PDU.

*Table 35. Process “Hammer Mill, Particle size<2mm, Forest residue”.*

| **Products and co-product** | |
| --- | --- |
| Hammer Mill, Particle size<2mm, Forest residue^a^ | 1 ton |
| **Materials/fuels** | |
| Electricity, medium voltage, at grid/US with US electricity U | 34.51 kWh |
| Grinder, Particle size<2mm, Forest residue | 1 ton |
| Conveyor belt, at plant/RER/I with US electricity U | 3.47E-05 m |

^a^ INL PDU.

*Table 36. Process “Preprocess, Pyrolysis, Forest residue”.*

| **Products and co-product** | |
| --- | --- |
| Preprocess, Pyrolysis, Forest residue^a^ | 1 ton |
| **Materials/fuels** | |
| Hammer Mill, Particle size<2mm, Forest residue | 0.25 ton |
| Grinder, Particle size<2mm, Forest residue | 0.75 ton |

^a^ INL PDU.

*Table 37. Process “Grinder, Particle size<1/4", Forest residue”.*

| **Products and co-product** | |
| --- | --- |
| Grinder, Particle size<1/4", Forest residue^a^ | 1 ton |
| **Materials/fuels** | |
| Conveyor belt, at plant/RER/I with US electricity U | 3.47E-05 m |
| Transport, truck | 72 tkm |
| Plant site storage | 0.1 ton |
| Electricity, medium voltage, at grid/US with US electricity U | 12.3 kWh |

^a^ INL PDU.

*Table 38. Process “Hammer Mill, Particle size<1/4", Forest residue”.*

| **Products and co-product** | |
| --- | --- |
| Hammer Mill, Particle size<1/4", Forest residue^a^ | 1 ton |
| **Materials/fuels** | |
| Electricity, medium voltage, at grid/US with US electricity U | 9.8 kWh |
| Grinder, Particle size<2mm, Forest residue | 1 ton |
| Conveyor belt, at plant/RER/I with US electricity U | 3.47E-05 m |

^a^ INL PDU.

*Table 39. Process “Preprocess, Pellet”.*

| **Products and co-product** | |
| --- | --- |
| Preprocess, Pellet^a^ | 1 ton |
| **Materials/fuels** | |
| Hammer Mill, Particle size<1/4", Forest residue | 0.15 ton |
| Grinder, Particle size<1/4", Forest residue | 0.85 ton |

*Table 40. Process “Cooling”.*

| **Products and co-product** | |
| --- | --- |
| Cooling^a^ | 1 ton |
| **Materials/fuels** | |
| Electricity, medium voltage, at grid/US with US electricity U | 0.34 kWh |
| Electricity, medium voltage, at grid/US with US electricity U | 0.56 kWh |

^a^Fantozzi, F., Buratti, C. 2010. Life cycle assessment of biomass chains: Wood pellet from short rotation coppice using data measured on a real plant. Biomass and Bioenergy, 34(12): 1796-1804.

*Table 41. Process “Power Plant, Biomass”.*

| **Products and co-product** | |
| --- | --- |
| Power Plant, Biomass ^a^ | 1,000 MJ |
| **Resources** | |
| Preprocess, Power Plant | 0.234 ton |
| Water, cooling, unspecified natural origin/m3 | 3.5 m^3^ |
| **Materials/fuels** | |
| Water, completely softened, at plant/RER with US electricity U | 6 kg |
| Water, decarbonised, at plant/RER with US electricity U | 150 kg |
| **Emissions to air** | |
| Carbon dioxide, biogenic | 585 g |
| Carbon monoxide, biogenic | 389 g |
| Nitrogen dioxide | 779 g |
| VOC, volatile organic compounds | 214 g |
| Particulates | 97 g |
| Sulfur dioxide | 389 g |

^a^Spath, P.L., Mann, M.K., Kerr, D.R. 1999. Life cycle assessment applied to electricity generation from renewable biomass & Life Cycle Assessment of Coal-fired Power Production (NREL). NREL/TP-570-25119.

*Table 42. Process “Pellet Mill, Forest residue”.*

| **Products and co-product** | |
| --- | --- |
| Pellet Mill, Forest residue^a^ | 1 ton |
| **Materials/fuels** | |
| Electricity, medium voltage, at grid/US with US electricity U | 50 kWh |
| Cooling | 1 ton |

^a^INL PDU.

*Table 43. Process “Pellet, distribution”.*

| **Products and co-product** | |
| --- | --- |
| Pellet, distribution^a^ | 1 ton |
| **Materials/fuels** | |
| Wheel Loader L150G | 1 ton |
| Transport, combination truck, diesel powered/US | 100 tkm |

^a^INL PDU.

*Table 44. Process “Pellet, combustion, Forest residue”.*

| **Products and co-product** | |
| --- | --- |
| Pellet, combustion, Forest residue^a^ | 1 kg |
| **Materials/fuels** | |
| Methane, biogenic | 0.035 g |
| Carbon monoxide, biogenic | 12.57 g |
| Carbon dioxide, biogenic | 1059 g |
| Ammonia | 0.002 g |
| Nitrogen dioxide | 0.643 g |
| Dinitrogen monoxide | 0.028 g |
| Sulfur dioxide | 4.226 g |
| Particulates | 0.063 g |
| **Waste treatment** | |
| Disposal, wood ash mixture, pure, 0% water, to sanitary landfill/CH with US electricity U | 0.033535 kg |

^a^ Brassard, P., Palacios, J.H., Godbout, S., [Bussières, D](http://www.ncbi.nlm.nih.gov/pubmed/?term=Bussières D%5BAuthor%5D&cauthor=true&cauthor_uid=24462881)., [Lagacé, R](http://www.ncbi.nlm.nih.gov/pubmed/?term=Lagacé R%5BAuthor%5D&cauthor=true&cauthor_uid=24462881)., [Larouche, J.P](http://www.ncbi.nlm.nih.gov/pubmed/?term=Larouche JP%5BAuthor%5D&cauthor=true&cauthor_uid=24462881)., [Pelletier, F](http://www.ncbi.nlm.nih.gov/pubmed/?term=Pelletier F%5BAuthor%5D&cauthor=true&cauthor_uid=24462881). 2014. Comparison of the gaseous and particulate matter emissions from the combustion of agricultural and forest biomasses. Bioresource Technology, 155: 300-306.

*Table 45. Process “Feller-Buncher*”.

| **Products and co-product** | |
| --- | --- |
| *Feller-Buncher* ^a^ | 1 *ton* |
| **Materials/fuels** | |
| Diesel, combusted in industrial equipment/US^b^ | 0.15 *gal* |
| Lubricant oil (1)^b^ | 0.002688 *gal* |

^a^ Greene, W.D., Biang, E., Baker, S.A. 2014. Fuel consumption rates of southern timber harvesting equipment. Proceeding: 37^th^ Council on Forest Engineering Annual Meeting, Moline, Illinois, USA;

^b^Ecoinvent 2.2.
